# Supplementary figures and images for: Mapping Staphylococcus aureus at Early and Late Stages of Infection in a Clinically Representative Hip Prosthetic Joint Infection Rat Model
Source: Microorganisms. 2024 Sep 14;12(9):1895. doi: 10.3390/microorganisms12091895 (PMC11433939; doi:10.3390/microorganisms12091895)

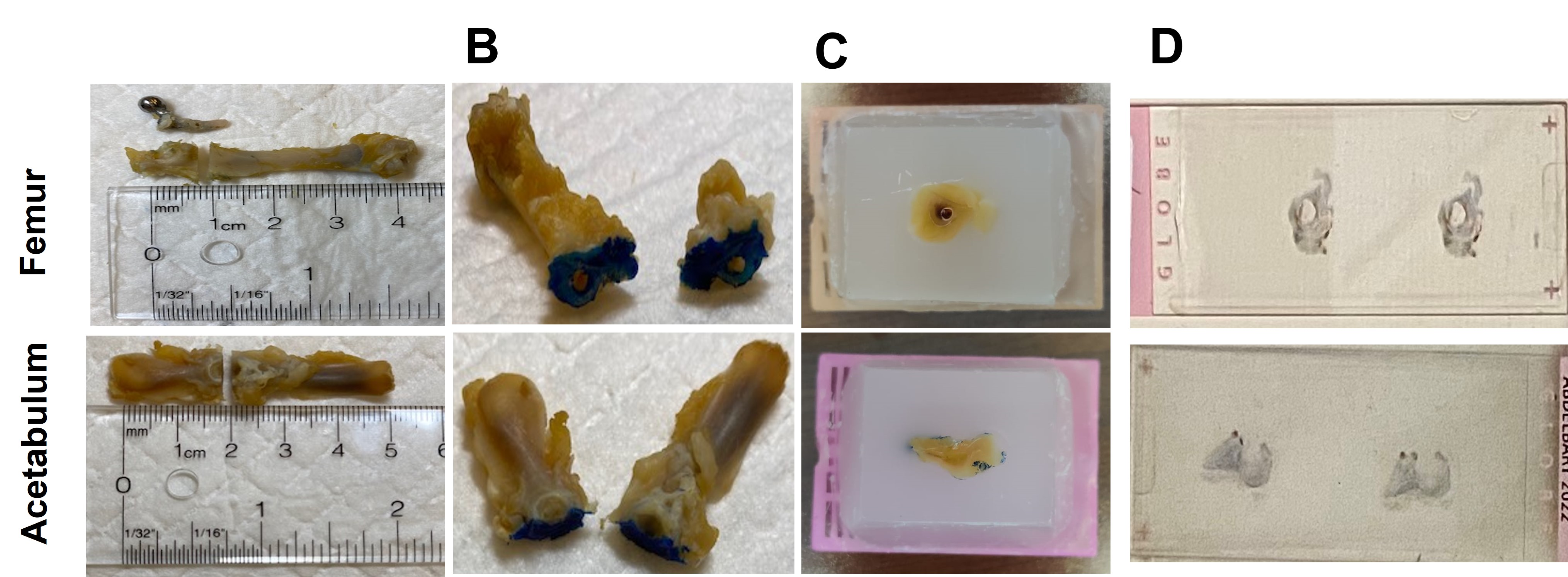

Supplement: Supplementary file 1 [file microorganisms-12-01895-s001.zip › Suplement Figure S2.jpg]

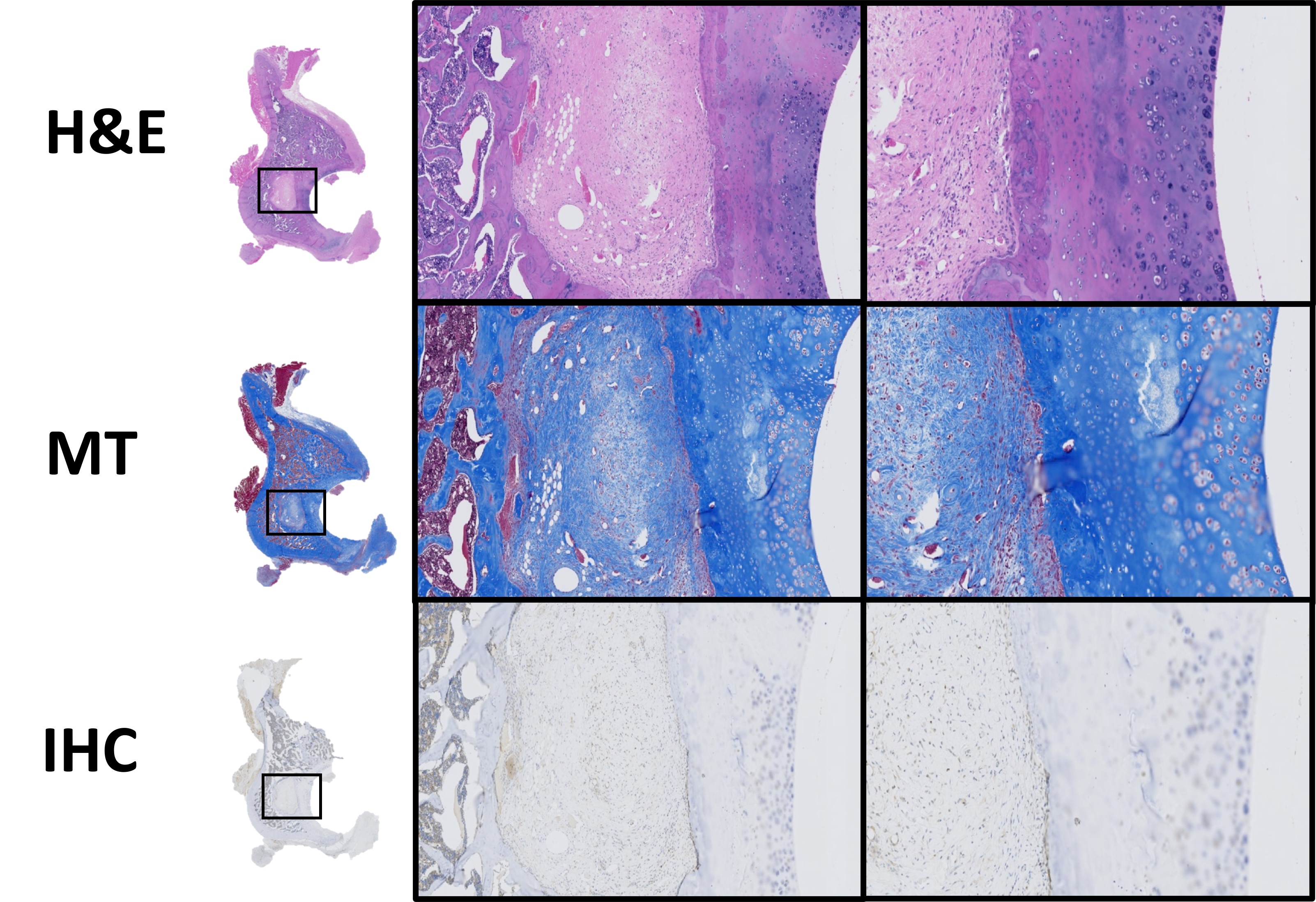

Supplement: Supplementary file 1 [file microorganisms-12-01895-s001.zip › Suplement Figure S3.jpg]

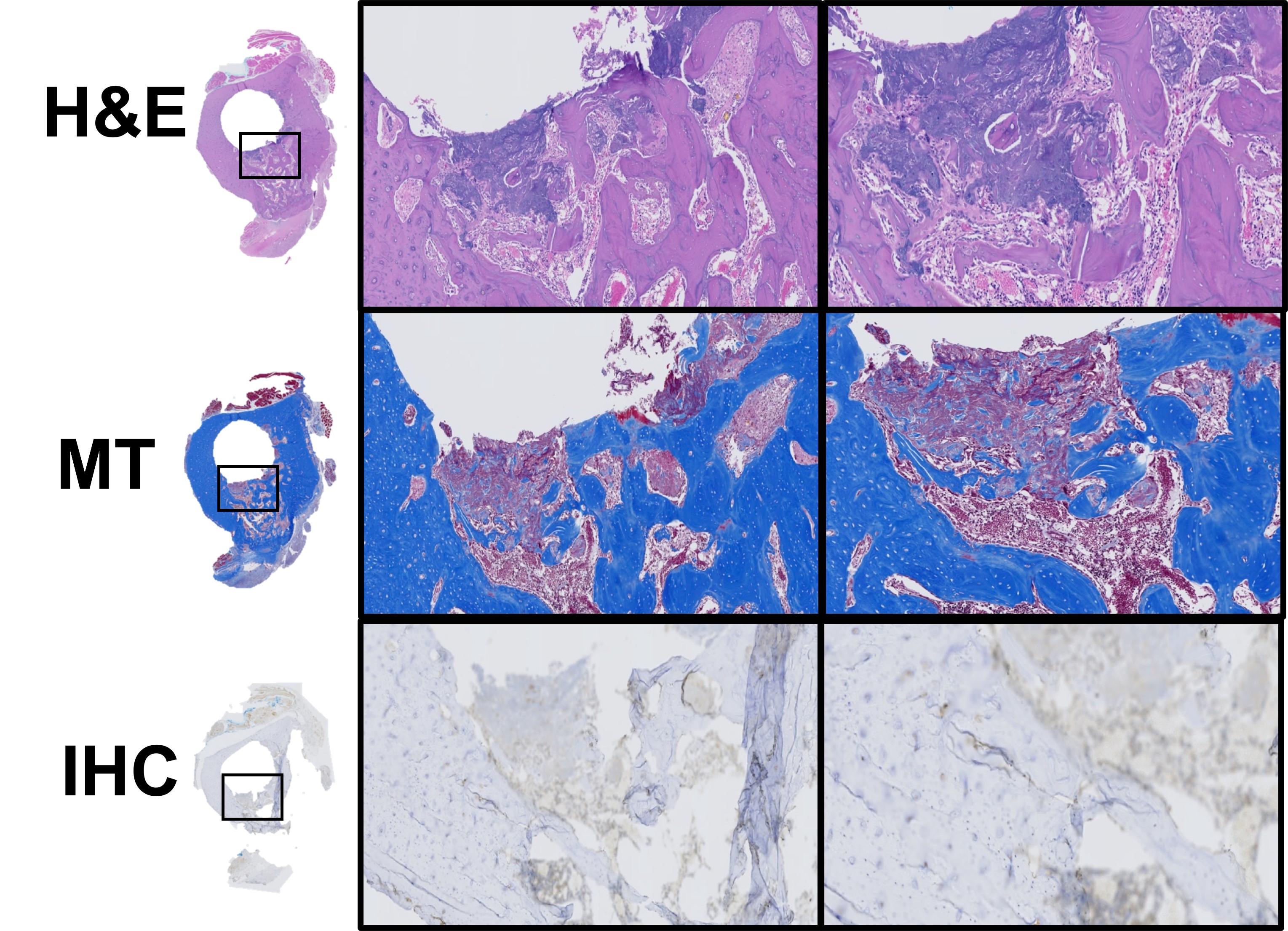

Supplement: Supplementary file 1 [file microorganisms-12-01895-s001.zip › Suplement Figure S4.jpg]

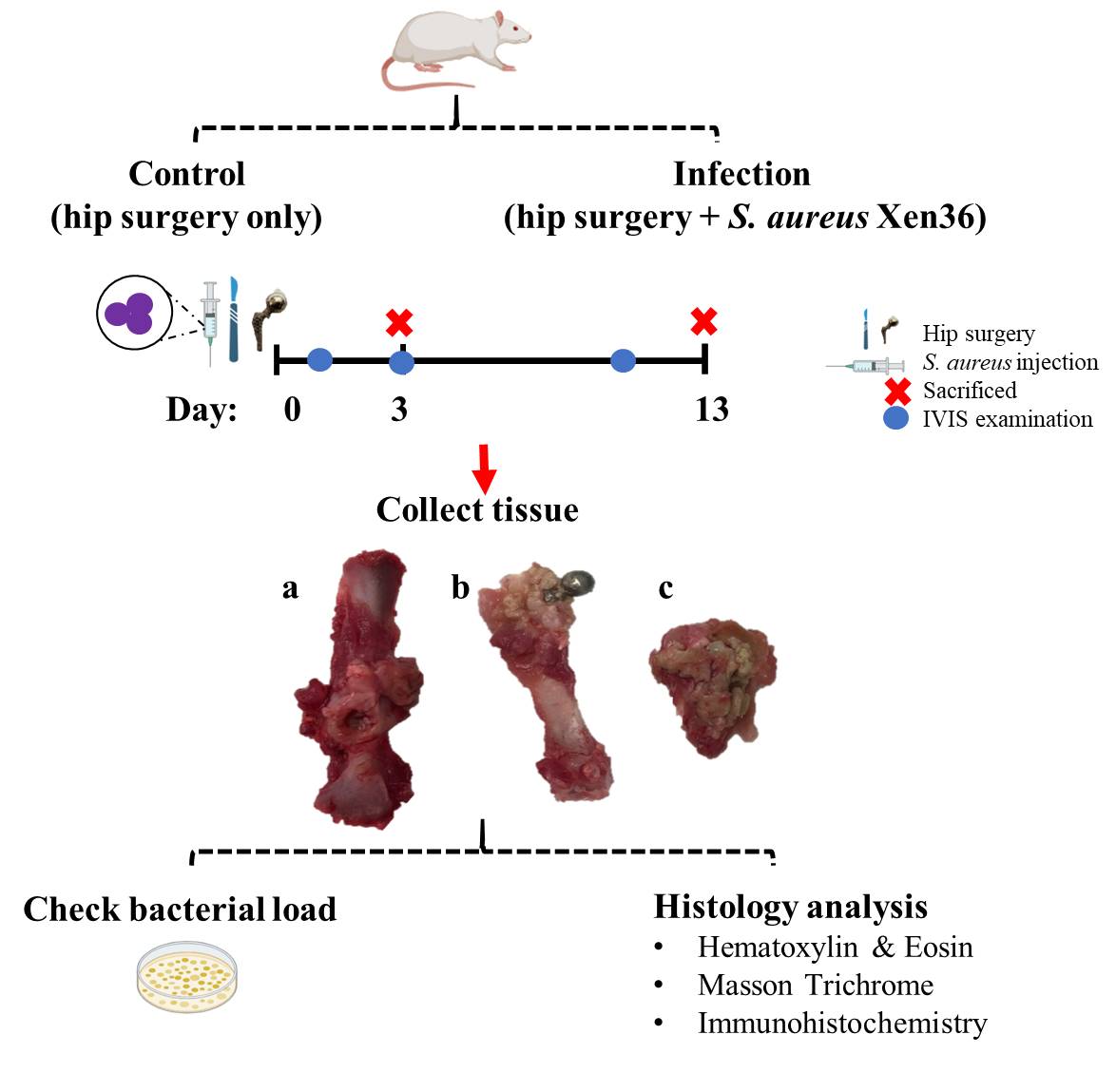

Supplement: Supplementary file 1 [file microorganisms-12-01895-s001.zip › Supplement Figure S1.jpg]
